# Grazing and Metabolism of *Euphausia pacifica* in the Yellow Sea

**DOI:** 10.1371/journal.pone.0115825

**Published:** 2015-02-17

**Authors:** Zhencheng Tao, Chaolun Li, Song Sun

**Affiliations:** 1 Key Laboratory of Marine Ecology and Environmental Sciences, Institute of Oceanology, Chinese Academy of Sciences, Qingdao, China; 2 Jiaozhou Bay Marine Ecosystem Research Station, Institute of Oceanology, Chinese Academy of Sciences, Qingdao, China; The Evergreen State College, UNITED STATES

## Abstract

Grazing and metabolism of *Euphausia pacifica* in the Yellow Sea were studied from September 2006 to August 2007. *Euphausia pacifica* is a selective-feeding omnivore and grazing rates among different months were monitored using a Coulter Counter and batch culture feeding experiments. *Euphausia pacifica* mainly grazed microzooplankton in August and September, which resulted in an increase in chlorophyll *a* concentration. Oxygen consumption rate of *E. pacifica* was 38.7–42.5 μmol O_2_ g^-1^ DW h^-1^ in March, which was four times higher than the oxygen consumption rates in September and December. The vigorous metabolism of *E. pacifica* in March consumed 3.1% of body carbon daily, which is likely related to its high reproduction and grazing rate. Respiration and metabolism of *E. pacifica* in September and December were similar and were lower. O:N ratio of *E. pacifica* was the highest (17.3–23.8) in March when spawning activity occurred and when food was abundant. The energetic source of *E. pacifica* during September and December was mostly protein from eating a carnivorous diet, including such items as microzooplankton. *Euphausia pacifica* was found in cold water at the bottom of the Yellow Sea in summer and autumn and maintained a low consumption status. O:N ratios of *E. pacifica* in March, September, and December were negatively correlated with SSTs and no significant correlation was found between O:N ratios and chlorophyll *a* concentration. Seawater temperature is clearly the most important parameter influencing the metabolism of *E. pacifica*.

## Introduction

As one of six important euphausiids species being commercially harvested, *Euphausia pacifica* is a large pelagic crustacean and is widely distributed in the North Pacific Ocean and adjacent coastal waters [1–5]. It is regarded as the key predominant euphausiid species in the Yellow Sea [6,7], where it constitutes more than 50% of the biomass of the large crustaceans (body length > 5 mm) throughout the year and nearly 80% in spring [8]. *Euphausia pacifica* is a key pelagic grazer [9] and the main food of many commercial fish in the Yellow Sea, especially adult anchovy [10,11]. It is also harvested for human consumption and as an additive for aquaculture feed [12]. In the Yellow Sea ecosystem, *E*. *pacifica* is regarded as one of the most important zooplankton species and functional group by the China-Global Ocean Ecosystem Dynamics (China-GLOBEC) project by virtue of its enormous abundance, large body size, and significant role in marine food chains.

The Yellow Sea is a relatively shallow (mean depth: 45.3 m, maximum depth: 140 m) and semi-enclosed marginal sea of the Pacific Ocean, where seasonal variations of whole seawater column temperatures range from 6°C to 28°C and surface temperature can be up to 28°C during the summer [13–15]. The spatial distribution of *E*. *pacifica* in the Yellow Sea is determined primarily by seawater temperature, with a tendency to inhabit cold water (8–16°C) [16,17]. As an oceanic species, *E*. *pacifica* has successfully lived and reproduced in the Yellow Sea region. The Yellow Sea bottom cold water (YSBCW) plays a very important role in the Yellow Sea ecosystem [18,19]. The YSBCW offers a cool-water refuge for *E*. *pacifica*, allowing it to survive during the summer and autumn when surface seawater temperatures are high [16].

In order to determine the role of *E*. *pacifica* in material flow and energy flux in the Yellow Sea ecosystem, it is essential to understand its grazing and metabolism. There is previous research into metabolism, production and growth of *E*. *pacifica* in the southern Sea of Japan, as well as in the western subarctic and northeastern Pacific Ocean [20–22]. Despite the importance of *E*. *pacifica* in the Yellow Sea ecosystem, these rates have not been reported previously. Several questions have not yet been answered: (1) What is the seasonal variability in grazing and metabolic rates of *E*. *pacifica* in the Yellow Sea? (2) Is temperature clearly the most important parameter influencing its metabolism and grazing rates? (3) What is the growth status of *E*. *pacifica* living in the YSBCW during summer and autumn?

Most studies on the grazing behavior of *E*. *pacifica* have been performed in the laboratory, using a diet of cultured phytoplankton and zooplankton [23–25]. Suh *et al*. studied the diet of *E*. *pacifica* calyptopes in the Yellow Sea by using scanning electron microscopy (SEM) to determine foregut contents [26]. Based on gut-content analysis and grazing experiments, many studies have suggested that *E*. *pacifica* is an omnivore [27,28] and can feed on a variety of phytoplankton [29] and microzooplankton [30,31], as well as suspended organic matter [32]. Kim *et al*. monitored the rates of oxygen consumption, ammonia excretion, O:N ratio (by atoms), and elemental composition of *E*. *pacifica* during the phytoplankton bloom season in the Oyashio region and also estimated daily ingestion and ammonia-N regeneration [21].

There is no systematic research on *E*. *pacifica* growth in the Yellow Sea; thus there is a need for more field investigations and experiments to improve our understanding of this important species. In the present study, we document the seasonal variation in grazing and metabolism of *E*. *pacifica* in the Yellow Sea, and examine the factors influencing its growth to better understand the ecological function of the YSBCW in *E*. *pacifica* growth and reproduction.

## Materials and Methods

### Ethics statement

No vertebrates were collected or used in our studies. No specific permissions were required for the described field studies. The studied area in the Yellow Sea is not privately owned or protected in any way and does not involve endangered or protected species.

### Field collections

**Fig 1:**
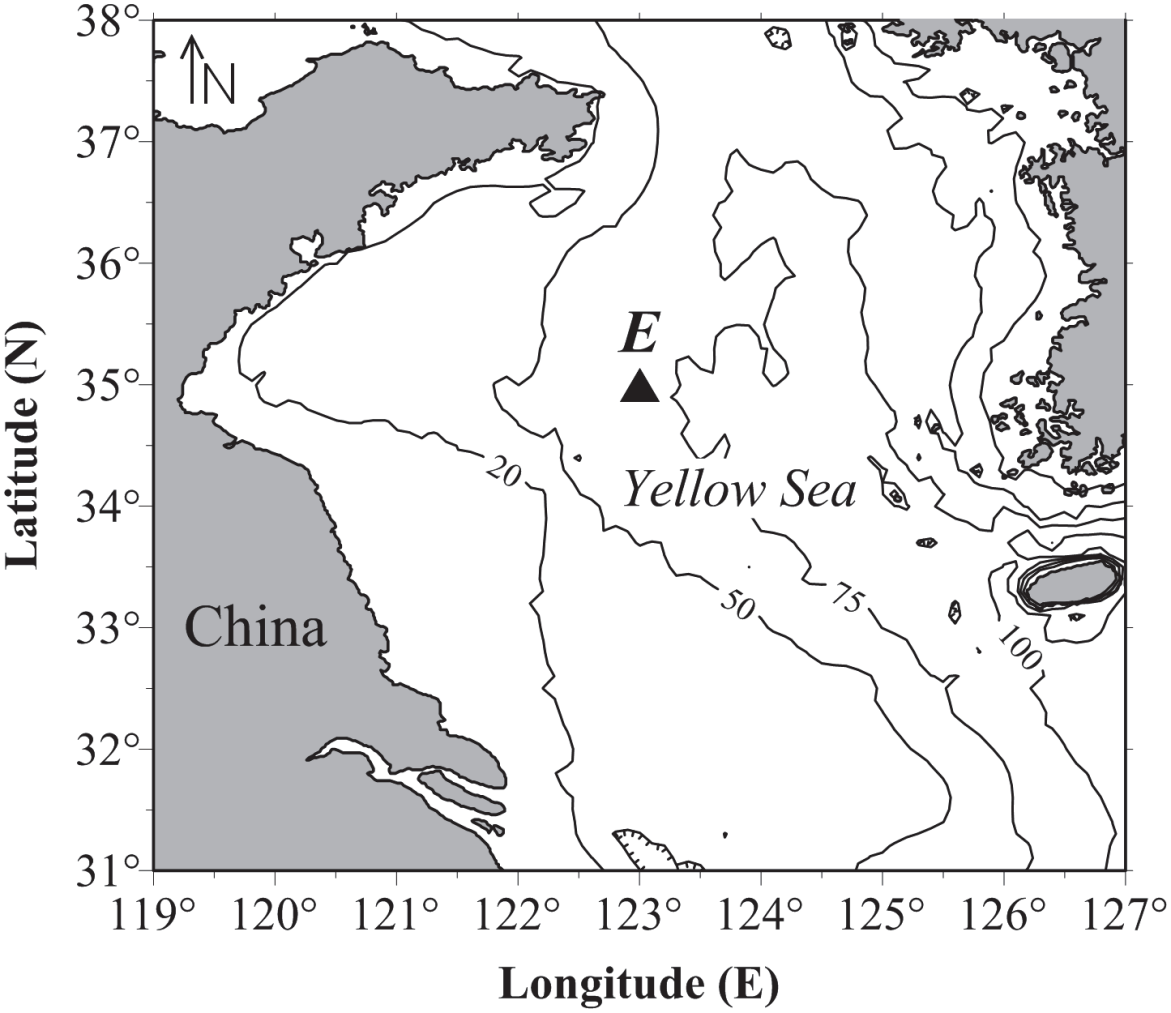
Location of the experimental station E in the Yellow Sea from September 2006 to August 2007. Twenty meter, 50 m, 75 m, and 100 m isobaths are shown.

Five grazing experiments and three metabolism experiments were conducted onboard the R.V. “Bei Dou” during five cruises from September 2006 to August 2007 in the Yellow Sea. The five cruises were conducted from September 18–28, 2006, October 15–31, 2006, December 20–31, 2006, March 16–25, 2007 and August 2–10, 2007. Unfortunately, due to bad weather and unforeseen circumstances during field surveys, the metabolism experiments were unable to be conducted in August and October. Fig. 1 shows the location of station E (123°E 35°N, depth: 74 m), which is located within the area of the YSBCW. Sampling at station E for field experiments was mainly conducted at night. A macrozooplankton net (conical net, diameter: 80 cm, mesh size: 500 μm) was towed from 2 m above the bottom to the surface at a rate of 0.7±0.1 m s^-1^ at night. The contents of the cod-end were transported to the laboratory in 20-L plastic buckets filled with unfiltered seawater. Adult *E*. *pacifica* used for experiments were actively swimming, apparently healthy and with no obvious damage. They were picked out with soup spoons and a group of 10 adults was placed in each 4-L bucket filled with 10°C (the *in situ* sampling condition temperature) seawater and kept in the dark for less than one hour, with an aim to minimize stress and to complete the preliminary tasks of the grazing and metabolic experiments during this period.

### Environmental factors

Temperature and salinity were measured using a CTD (Sea-Bird Electronics, SBE 25). Samples for chlorophyll *a* (Chl *a*) analysis were collected at the surface and at 5, 10, 20, 30, 50 and 72 m layers with 5-L Niskin bottles. The standard fluorometric methodology was used for measuring chlorophyll. Five hundred mL of seawater was filtered through a 25-mm diameter Whatman GF/F glass fiber filter (pore size 0.70 μm) and immediately stored in a freezer at -20°C. After field survey (less than one month), the filters were measured in the laboratory. Chl *a* was extracted from the filters using 90% acetone for 24 hours in a refrigerator (≤4°C) and then the fluorescence of Chl *a* was measured with a Turner Designs Fluorometer 7200.

### Grazing experiments

In industrial applications historically it has been common practice to report particle ‘size’ in linear terms, and so the volume of a particle is converted into an equivalent spherical diameter (ESD). The “particle size” is the diameter of a sphere whose volume is equal to that of the particle. Coulter Counter is the fastest and most efficient method to measure changes in particle ESD size spectrum and volume [33,34], which has been used to study the ingestion of zooplankton, especially copepods [35–37]. In our studies, a Coulter Counter (BECKMAN Multisizer 3) was used for the first time for a grazing analysis of *E*. *pacifica*. The food particle size spectra of *E*. *pacifica* grazing experiment samples were monitored by use of a Coulter Counter with a 100-μm aperture.

In order to determine the grazing rates of *E*. *pacifica* and the food environment in the field, 24 hours feeding experiments were conducted. Seawater for the experiment was collected from the water column layer with the highest Chl *a* and filtered with a 200-μm mesh. At the beginning of the experiments, 1 L seawater samples used for grazing experiments were fixed with 1% acid Lugol’s iodine and stored at < 4°C in the dark for analysis of ciliates, which were concentrated and examined by the Utermöhl method [38]. Ciliates were counted in the laboratory, and the length and diameter of each cell was measured. The cell size of each ciliate was converted to ESD and its volume was estimated using appropriate geometric shapes. The carbon:volume ratio (C:V) used to calculate ciliate biomass was 0.19 pg C μm^-3^ [39].

Firstly, the gut fullness of *E*. *pacifica* was checked and it was confirmed that the gut was empty or gut fullness was lower than 10%. Five adult *E*. *pacifica* were added to each 1.5-L polycarbonate bottle, which was filled with the collected seawater. The gross and size-fractionated (0.70–2, 2–5, 5–20, and >20 μm) phytoplankton Chl *a* concentration samples of seawater were used as the initial Chl *a* concentrations. Three additional grazing containers filled with 1.5 L of seawater were used as controls with no added *E*. *pacifica*. Nitrogen and phosphorus from zooplankton excretion could promote phytoplankton growth. A nutrient mixture (the final concentrations of bottles were 10 μmol L^-1^ NaNO_3_, 10 μmol L^-1^ Na_2_SiO_3_, and 1 μmol L^-1^ NaH_2_PO_4_) was added to all bottles to override the effect of differing amounts of zooplankton excretion among the experiments [40]. Three replicates and three control bottles were used. All experiments were conducted in a temperature-controlled (10±0.5°C) incubator in the dark for 24 hours. The bottles were inverted gently every 6 hours during incubation to keep the phytoplankton suspended. At the end of the experiment, the samples were checked for dead *E*. *pacifica*, but none were found. Then 1000 mL of seawater from each bottle was filtered for gross and for size-fractionated phytoplankton Chl *a* concentrations. Finally, 150 mL of seawater from each bottle (including initial, control, and treatment) was preserved in 5% buffered formalin seawater solution to determine the cell/particle composition.

### Metabolic experiments

The seawater used for the metabolism experiments was similar to that used in the grazing experiments. To eliminate the effect of other plankton, the water was filtered first using a 20-μm mesh and then with a 0.45-μm pore size Millipore filter, leaving no food for *E*. *pacifica* was left in the culture bottles. The seawater was aerated for 4 hours in a 10±0.5°C constant temperature incubator. Five adult *E*. *pacifica* were placed into a 500-mL brown glass solution bottle, which had been filled with the well-oxygenated seawater. Then the bottle was covered with a rubber stopper and paraffin wax to ensure that no air bubbles remained. Three parallel bottles and three control bottles without *E*. *pacifica* were run at the same time. All bottles were put in a dark constant temperature incubator at 10±0.5°C for 24 hours. The initial dissolved oxygen (IDO) and final dissolved oxygen (FDO) of the seawater were determined by the Winkler titration method [41]. The oxygen consumption rate (OCR, μmol O_2_ g^-1^ DW h^-1^) of *E*. *pacifica* was calculated from the IDO and FDO. Meanwhile, at the beginning and end of the metabolism experiment, the ammonia nitrogen concentrations of the seawater were measured with a San^+^ automated wet chemistry analyzer (Skalar, Dutch) to calculate the ammonia-nitrogen excretion rates (NER, μmol NH_3_ g^-1^ DW h^-1^) of *E*. *pacifica*. Finally, *E*. *pacifica* was rinsed briefly with a small amount of distilled water to remove salts, blotted on filter paper and frozen at -20°C. At the land laboratory, the frozen *E*. *pacifica* samples were dried in an oven at 60°C for 24 hours to obtain their dry weight (DW) and body carbon content which was measured with a PE240C elemental analyzer (PerkinElmer, USA).

The atomic O:N ratio was calculated with OCR and NER. The carbon consumption rate (CCR) was considered as the energy index of zooplankton routine metabolism. The relationship between CCR and OCR was expressed as:

CCR = OCR × RQ × 12

In the formula, the respiratory quotient (RQ) indicated the molar ratio between CO_2_ excretion and O_2_ consumption. The RQ of ocean zooplankton is 0.72 (O:N ratio greater than 24 indicates lipid-oriented-metabolism) or 0.97 (O:N ratio less than 24 indicate protein-oriented metabolism) [42,43]. Daily body carbon consumption (% d^-1^) was calculated with CCR and body carbon of DW.

After each experiment was completed, the total length (TL, mm), from the curve of carapace behind the eye to the tip of the telson, of each *E*. *pacifica* was measured by using a binocular dissecting microscope with a calibrated eyepiece reticle micrometer [44].

### Data analysis and statistical methods

The sampling station map (Fig. 1) was generated using Golden Software Surfer V8.0. Data analyses were performed with SPSS V16.0 software and correlations were evaluated statistically using a Spearman rank correlation. The respiration, excretion rates of *E*. *pacifica* and the body characteristics (DW, body carbon contents) during the different months were compared using one-way ANOVA followed a *post hoc* Scheffe´ test.

## Results

### Physical environment and chlorophyll *a*

**Fig 2:**
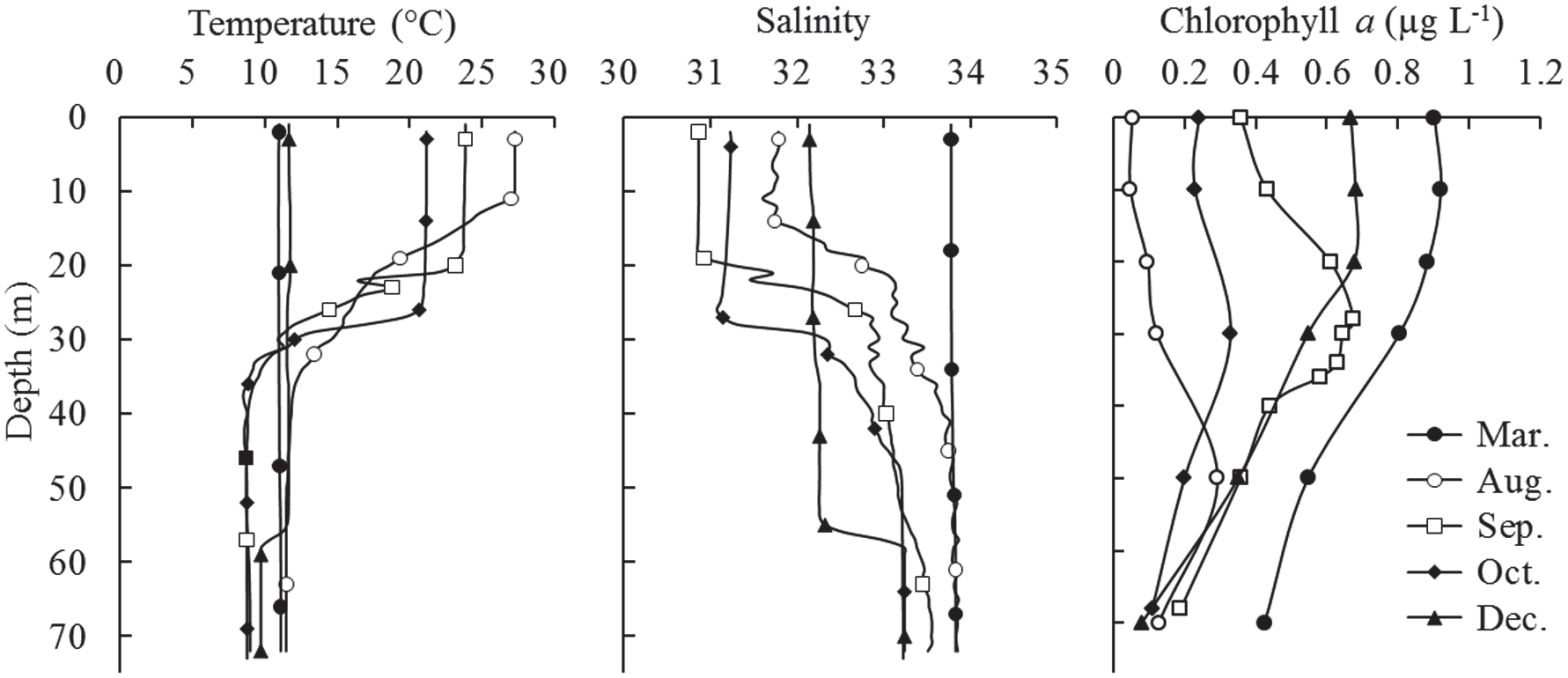
Vertical distributions of temperature, salinity and chlorophyll *a* concentrations at station E during five cruises from September 2006 to August 2007 in the Yellow Sea.

The vertical profiles of seawater temperature, salinity, and Chl *a* concentrations at the experimental station during five cruises are shown in Fig. 2. The average seawater temperature of the whole water column in March, August, September, October, and December was 11.0, 15.9, 13.9, 13.5, and 11.2°C, respectively. A thermocline occurred in August, September and October. It was the thickest in August (12–36 m depth) and the thinnest in October (27–35 m depth). T-S diagrams of 20 oceanographic stations illustrated seasonal variation of water masses in the study area and the occurrence of the YSBCW in summer and autumn [16]. The cold water mass near the bottom, the YSBCW was evident in those months. The surface seawater temperatures (SSTs) were higher than 20°C at that time. The highest SST (27.3°C) occurred in August 2007. All bottom seawater temperatures (BSTs) were lower than 12°C during five cruises. The largest difference between surface and bottom seawater temperatures (15.8°C) occurred in August. The seawater salinity increased from the surface to bottom of the water column from August to October. When the water column was vertically mixed in March and December, surface and bottom water temperature and salinity were similar and stable and the differences in seawater temperatures were less than 2.0°C.

The Chl *a* concentration reached a maximum in March for all five cruises. It decreased from the surface to the bottom of the water column during March and December. The maximum Chl *a* concentration occurred in the 10-m layer in March. In August, September, and October, the Chl *a* concentrations were relatively lower, with the maximum value (0.92 μg L^-1^) in the thermocline layer. The average Chl *a* concentration in August was lowest at only 0.12 μg L^-1^.

### Food composition changes in grazing experiments and total length variation of *Euphausia pacifica*

**Fig 3:**
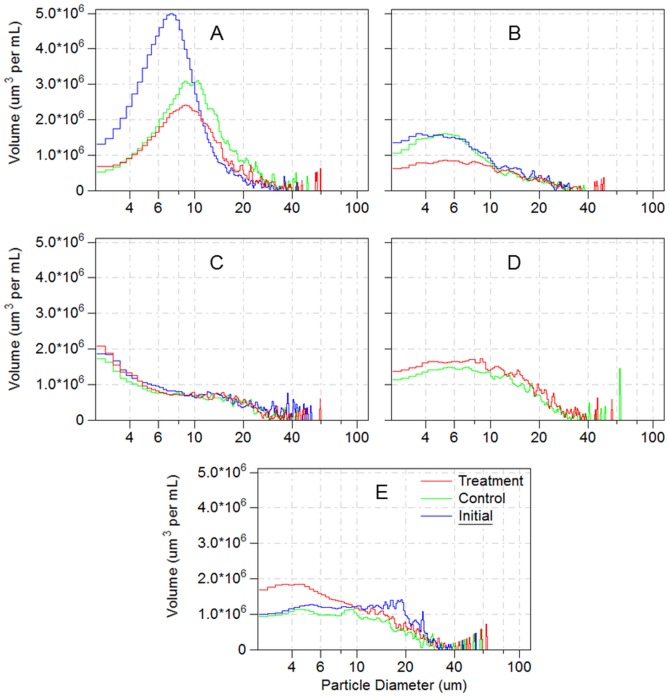
Volume variations of different ESD size particles in *Euphausia pacifica* grazing experiments on gross food samples in the Yellow Sea. A, March 2007; B, August 2007; C, September 2006; D, October 2006; E, December 2006.

Variations in particle size spectra and gross volume in the grazing experiment samples in the Yellow Sea during five months are shown in Table 1. Food composition changes, measured as the particle size spectrum and volume in the grazing experiments, during five cruises are shown in Fig. 3. The particles in the experimental seawater could be composed of phytoplankton, microzooplankton, and suspended organic matter, which could be grazed by *E*. *pacifica*. Unfortunately, initial samples were lost from October 2006. During the other four months, the initial food volume changed with season; specifically values were 120.4*10^6^ μm^3^ mL^-1^ in March, 54.6*10^6^ μm^3^ mL^-1^ in August, 69.2*10^6^ μm^3^ mL^-1^ in September, and 87.2*10^6^ μm^3^ mL^-1^ in December. The mean particle size in March (9.6±6.0 μm) was the smallest of the five months. The food source (mainly phytoplankton) was most abundant in March, with particles smaller than 20 μm ESD constituting 93.6% of the volume of gross food.

**Table 1:** Variations of particle size spectra (mean±SD, μm) and gross volume (*10^6^ μm^3^ mL^-1^) in *Euphausia pacifica* grazing experiments in the Yellow Sea during five months.

	March	August	September	October	December
**spectra (initial)**	9.6±6.0	10.8±6.7	18.9±13.7	na	15.2±8.8
**spectra (control)**	14.1±8.5	10.7±7.2	14.9±10.2	15.9±13.2	15.2±11.4
**spectra (treatment)**	14.5±11.3	14.5±11.0	16.2±12.9	14.7±10.0	15.6±13.0
**gross volume (initial)**	120.4	54.6	69.2	na	87.2
**gross volume (control)**	120.0	48.8	53.0	80.5	60.3
**gross volume (treatment)**	91.3	40.2	58.9	95.2	85.0

na: no data (samples were lost).

After 24 hours, the change in particle size spectra in March was larger than in other months. The mean particle size increased from 9.6±6.0 to 14.1±8.5 in the control and 14.5±11.3 in the treatment. No obvious changes in mean size spectra were found between controls and treatments during March, October, and December. Compared to the control, larger size particles in treatments with *E*. *pacifica* cultured for 24 hours increased greatly in August. Smaller sized food particles of treatments were ingested in August and September (Table 1). The total food volume of the control was 120.0*10^6^ μm^3^ mL^-1^ in March, but decreased in August and increased in both September and December. Furthermore, the size composition of particles obviously changed. In comparison to the control, the volume of gross food samples of the treatments decreased after *E*. *pacifica* feeding in both March and August. Particles between 5–25 μm ESD were mainly grazed in March and particles between 2–11 μm ESD were mainly grazed in August. The changes in gross food particle size composition of the treatments during September, October and December were opposite to those in March and August. The gross food volumes of the treatments during September, October, and December were higher than their control groups and increased 5.9, 14.7, and 25.3*10^6^ μm^3^ mL^-1^, respectively. The changes in particle size spectra in the treatments showed that larger particles/cells decreased and smaller ones increased.

**Fig 4:**
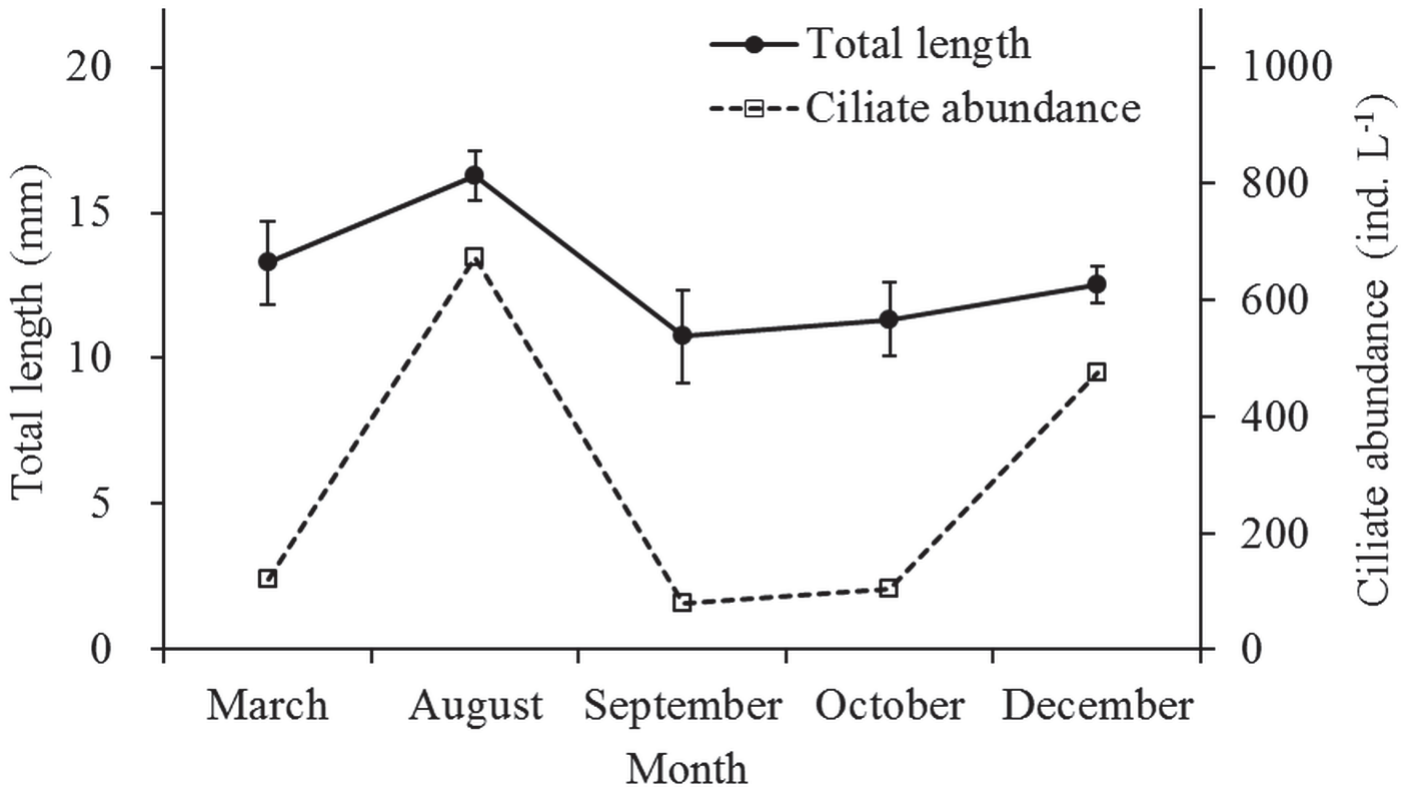
Ciliate abundance and *Euphausia pacifica* total length variation during five cruises from September 2006 to August 2007 in the Yellow Sea.

Ciliate abundance in the grazing experiment seawater was high (672 ind. L^-1^) in August and low (about 80–120 ind. L^-1^) in March, September, and October (Fig. 4). Ciliates of 15–30 μm ESD dominated numerically during the five months. According to the C:V and ciliate volume, ciliate biomass of each grazing experiment bottle was 0.49 μg C in March, 2.72 μg C in August, 0.32 μg C in September, 0.43 μg C in October, and 1.92 μg C in December. The TL of *E*. *pacifica* was largest (16.27±0.83 mm) in August and smallest (10.75±1.60 mm) in September. The trend in TLs of *E*. *pacifica* was similar to the pattern in ciliate abundance and indeed TL of *E*. *pacifica* were positively correlated with ciliate abundance (Correlation coefficient = 0.900, N = 5, P<0.05) (Fig. 4).

### Feeding on phytoplankton by *Euphausia pacifica*

**Fig 5:**
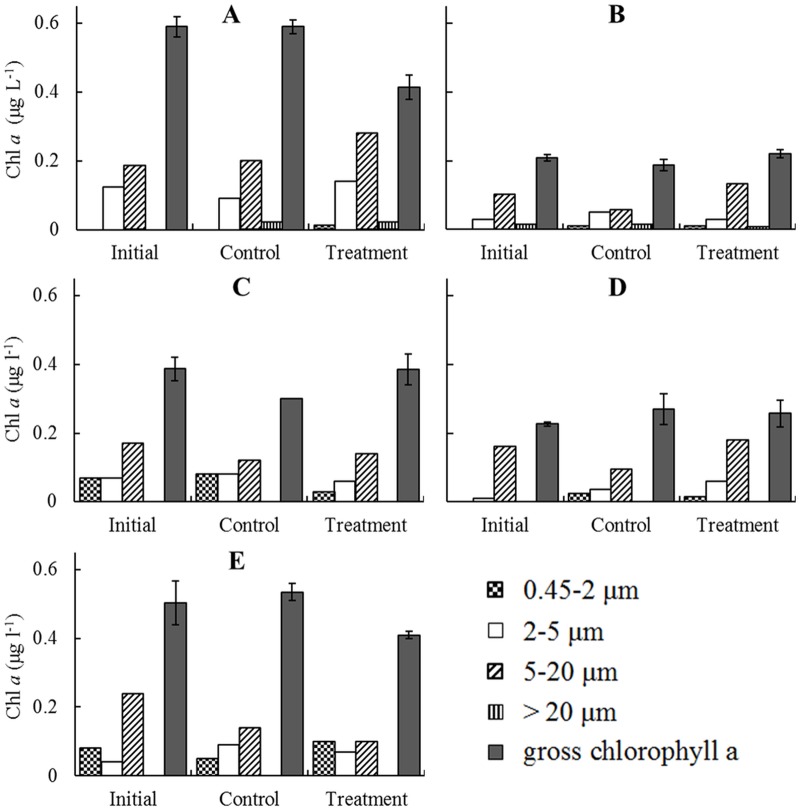
Variation in chlorophyll *a* concentrations in *Euphausia pacifica* grazing experiments from the Yellow Sea. A: March 2007, B: August 2007, C: September 2006, D: October 2006, E: December 2006.

The gross Chl *a* concentration in the treatment was about 30% lower than in the initial concentration in March; however, all size-fractionated Chl *a* increased between initial and final concentrations (Fig. 5A). Although the Chl *a* concentration in September 2006 was twice that in August 2007, the pattern of variation in Chl *a* concentrations in those months was similar. Compared to the control, mainly 0.45–2 μm and 2–5 μm size-fractionated Chl *a* were ingested, but the gross Chl *a* concentrations of the treatment increased after 24 hours of incubation during August and September. In other words, the increase in 5–20 μm size-fractionated Chl *a* concentration caused the increase in gross Chl *a* concentration of treatments relative to the controls (Fig. 5B and Fig. 5C). Initial Chl *a* concentration in October was lower than in September. Size-fractionated Chl *a* larger than 20 μm was not detected in the initial, control and treatment tests. The 5–20 μm size-fraction contributed to most of the total biomass of Chl *a*. The gross Chl *a* concentration of the treatment group was not significantly changed after *E*. *pacifica* grazing. Compared to the control, the 2–20 μm size-fraction increased with *E*. *pacifica* grazing (Fig. 5D). In December, Chl *a* concentrations of the initial, control, and treatment groups were all more than 0.40 μg L^-1^ and were higher than in October. Compared to the initial Chl *a* concentration (average: 0.50 μg L^-1^), the average Chl *a* concentration of the control was slightly higher (0.54 μg L^-1^), but the treatment average was much lower (0.41 μg L^-1^). Gross Chl *a* concentration was significantly consumed in the *E*. *pacifica* grazing treatment. After 24 hours, the 2–20 μm size-fraction Chl *a* in the treatment group decreased, but the 0.45–2 μm size-fraction Chl *a* in treatment increased to twice that of the control (Fig. 5E).

The sum of the size-fractionated Chl *a* concentrations was not equal to the gross Chl *a* concentration in any of the five months, especially in March. But the variation in different size-fractionated Chl *a* concentrations showed changes with treatment with *E*. *pacifica* grazing. In August and September, Chl *a* concentration increased after *E*. *pacifica* grazing, which suggests that the grazing rate of *E*. *pacifica* on phytoplankton was relatively low. In contrast, Chl *a* concentrations of the treatments in December and March significantly decreased. If phytoplankton was the only food for *E*. *pacifica*, the grazing rate of *E*. *pacifica* was 0.056 and 0.079 μg Chl *a* ind.^-1^ d^-1^ in December and March, respectively.

### Oxygen consumption, ammonia excretion and daily carbon demand of *Euphausia pacifica*

There was no statistical difference in the respiration and excretion rates of *E*. *pacifica* among parallel experiments (one-way ANOVA). Oxygen consumption rates of *E*. *pacifica* in September and December were similar (9.1±4.2 and 11.5±7.6 μmol O_2_ g^-1^ DW h^-1^) and lower than in March (40.9±2.0 μmol O_2_ g^-1^ DW h^-1^). NER of *E*. *pacifica* was lowest in March (4.2±0.8 μmol NH_3_ g^-1^ DW h^-1^). Using OCRs and NERs during three cruises, the atomic O:N ratios of *E*. *pacifica* in September, December 2006 and March 2007 were 1.6–2.7, 2.0–5.1, and 17.3–23.8, respectively (Table 2).

Using the formula for the relationship between CCR and OCR, CCRs of *E*. *pacifica* during three cruises were 62.9±3.1 (March), 11.2±5.2 (September), and 10.6±7.0 (December) μg C ind.^-1^ d^-1^, respectively. Average DWs of *E*. *pacifica* in March, September and December were 5.5, 4.4, and 3.3 mg ind.^-1^, respectively. The body carbon contents in those three months were 42.4%, 45.5%, and 44.7% of DW, respectively, meaning that *E*. *pacifica* stored more lipids in March than in September and December. The percentages of body carbon consumed daily by *E*. *pacifica* in those months were (3.1±0.2)%, (0.8±0.4)%, and (0.5±0.3)%, respectively (Table 2). O:N ratios of *E*. *pacifica* in March, September and December were negatively correlated with SSTs (Correlation coefficient = -1.000, N = 3, P<0.01) and no significant correlation was found between the O:N ratios and chlorophyll *a* concentrations (Correlation coefficient = 0.866, N = 3). Thus seawater temperature is clearly the most important parameter influencing the metabolism of *E*. *pacifica*.

**Table 2:** Metabolic parameters of *Euphausia pacifica* in the Yellow Sea in September 2006, December 2006, and March 2007.

	March	September	December
**OCR**	38.7–42.5	4.6–12.8	4.2–19.3
**OCR**	40.9±2.0	9.1±4.2	11.5±7.6
**NER**	3.3–4.9	5.6–9.6	4.2–7.6
**NER**	4.2±0.8	7.2±2.1	5.6±1.8
**O:N ratio**	17.3–23.8	1.6–2.7	2.0–5.1
**Average DW**	5.5±0.7	4.4±0.8	3.3±0.2
**Body carbon content (%)**	42.4±0.3	45.5±1.9	44.7±1.7
**CCR**	62.9±3.1	11.2±5.2	10.6±7.0
**Daily body carbon consumption (% d^-1^)**	3.1±0.2	0.8±0.4	0.5±0.3

OCR: oxygen consumption rate (μmol O_2_ g^-1^ DW h^-1^).

NER: ammonia-nitrogen excretion rate (μmol NH_3_ g^-1^ DW h^-1^).

DW: dry weight (mg ind.^-1^).

CCR: carbon consumption rate (μg C ind.^-1^ d^-1^).

## Discussion

### Grazing activity and rates of *Euphausia pacifica* in the Yellow Sea

*Euphausia pacifica* is a filter-feeding zooplankton that collects food from flowing water into a “food basket”, which is then put into its mouth with rapidly beating thoracic appendages [45]. Ohman found that *E*. *pacifica* was a selective-feeding omnivore in experiments with diatoms and copepods [24]. *Euphausia pacifica* cannot directly and effectively graze small components in the food web, such as bacteria. Dilling *et al*. found that *E*. *pacifica* could consume many types of marine snow [32], which were composed of coagulated small particles such as phytoplankton and fecal pellets, or directly formed by gelatinous zooplankton as discarded mucous feeding structures [46]; the grazing rates on marine snow did not vary with food quality [32]. According to our studies from September 2006 to August 2007, we confirmed that *E*. *pacifica* in the Yellow Sea was an omnivore and could select a diet based on food conditions, which agrees with the former studies.

The efficiency of primary production transfer to higher trophic levels is determined by grazing pressure of zooplankton on phytoplankton. Since the 1970s, the grazing pressure of meso- and microzooplankton on phytoplankton have been studied using batch culture feeding experiments [47], gut pigment [48], and dilution techniques [49]. Kang analyzed the gut-contents of 747 *E*. *pacifica* adults in the Yellow Sea and found that their diets included diatoms (primary food), dinoflagellates, ciliates, lamellibranches, and copepods [45]. Tintinnids are also frequently found in the gut contents of *E*. *pacifica* [30]. The batch culture feeding method was used in our grazing experiments. Carpenter *et al*. found that increased piscivore biomass caused decreased zooplanktivore biomass, increased herbivore biomass, and decreased phytoplankton biomass and defined this phenomenon as a cascading trophic interaction [50].

If we assume that phytoplankton was the only food supply and *E*. *pacifica* was the only grazer in the culture bottle, the grazing rates of *E*. *pacifica* during March and December were 0.079 and 0.056 μg Chl *a* ind.^-1^ d^-1^, respectively. The conversion ratio of phytoplankton biomass carbon to Chl *a* was 93 μg μg^-1^ in the outer Jiaozhou Bay region which is adjacent to the Yellow Sea [51], and was 92.8 μg μg^-1^ in the East China Sea [52]. We can calculate using the ratios that the maximum daily grazing on phytoplankton by *E*. *pacifica* during March and December were 7.35 and 5.21 μg C ind.^-1^ d^-1^, respectively. Our results are mostly lower than other studies which showed higher phytoplankton amounts and Chl *a* concentrations. The ingestion rate of *E*. *pacifica* on the marine snow in the Santa Barbara Channel was 9–15 μg C euphausiid^-1^ h^-1^ [32]. Du and Peterson found that the ingestion rates of *E*. *pacifica* in the coastal upwelling zone off Oregon ranged from 4.8 to 16.0 μg C euphausiid^-1^ h^-1^, that larger types of ciliates and dinoflagellates were fed upon preferentially and that diatoms were consumed almost exclusively during blooms associated with summer upwelling events [22]. Nakagawa *et al*. fed *E*. *pacifica* with ciliates, especially naked ciliates, in laboratory and field experiments and found that *E*. *pacifica* ingested cultured *Strombidium conicum* at rates of 0.04 to 0.07 μg C at high concentrations when they were given as the only prey [31]. They concluded that *E*. *pacifica* ingested naked ciliates and therefore played a role in linking microbial food webs to the classical grazing food chain. The ciliate community was found to be dominated by aloricate ciliates, accounting for 67–97% of the total abundance of ciliates in the Yellow Sea [53]. The ciliate biomass in bottles of grazing experiments was 0.49 in March, 0.32 in September, and 1.92 in December in μg C per bottle. The carbon consumption rates of *E*. *pacifica* during these three months were 59.5–65.3, 5.6–15.7 and 3.9–17.8 μg C ind.^-1^ d^-1^, respectively. Therefore, phytoplankton and ciliate food sources cannot meet *E*. *pacifica* growth and reproduction demands in March, September, and December and *E*. *pacifica* must graze on other microzooplankton and suspended particles.

Zhang *et al*. found that microzooplankters were the main grazers of primary production in the Yellow Sea and play important roles in the marine pelagic system [54]. The species of phytoplankton and microzooplankton were not identified and counted in our grazing experiments; instead, particle/cell size spectra of the seawater were measured with a Coulter Counter. Data in March indicated that 5–25 μm ESD particles/cells were mainly ingested by *E*. *pacifica* and microzooplankton, which did not concur with the changes in 5–20 μm size-fractionated Chl *a* concentrations. To the contrary, smaller particles/cells (<11 μm ESD) were primarily consumed in August. During September, October, and December, food components less than 25 μm ESD increased and were the highest in December. Both Coulter Counter and batch culture feeding methods in the grazing experiments come to a common conclusion that microzooplankton (e.g. ciliates, etc.) should be the first food of *E*. *pacifica*, especially during times of low Chl *a* concentrations in summer and autumn.

### Metabolism of *Euphausia pacifica* in the Yellow Sea

Recently, with new and improved methods, studies on the physiology and biochemistry of *E*. *pacifica* have greatly advanced. Respiration and metabolism of euphausiids are related to age and body-weight [55], swimming speed [56], and environmental factors such as temperature, oxygen concentration, and food [20,21,57–59]. The physiology and metabolism of *E*. *pacifica* in Puget Sound, including carbon and nitrogen budgets, respiration, and growth at 8°C and 12°C were studied in laboratory conditions [60,61].

Respiration rates can be used to estimate the amount of food needed to balance metabolism [59,62]. The routine metabolisms of *E*. *pacifica* were determined in these experiments and are related to growth and productive stage. Metabolism of *E*. *pacifica* in September and December were similar, but significantly lower than in March. *Euphausia pacifica* were vigorous in March and consumed 2.7% of body carbon daily, which was closely correlated to reproductive activity and high grazing rate. The average brood size was 71.33 eggs female^-1^ in the southern Yellow Sea in March 2007 [16]. The maximum daily body carbon consumption of *E*. *pacifica* in the coastal upwelling zone off Oregon was 23% at the highest food biomass (700 μg C L^-1^) and the averaged ratio was 4% over a carbon range of 50–200 μg C L^-1^ [22]. Ross found that *E*. *pacifica* could use 20% of body carbon daily when the prey *Thalassiosira weissflogii* exceeded 350 μg C L^-1^ [60]. Our results are much lower than in the former studies, which is mostly caused by the lower food concentration.

The atomic O:N ratio is regarded as a direct and effective tool to describe zooplankton metabolism [63]. The O:N ratio shows what biochemical component of the body is used as an energy source and reflects the relative utilization of protein in energy metabolism. A high rate of protein catabolism relative to lipid and carbohydrate catabolism results in a low O:N ratio, which generally indicates a stressed condition, such as starvation. An O:N ratio over 24 indicates a lipid-dominated catabolism [63–65].

From March through April, O:N ratios for *E*. *pacifica* in the Oyashio region fluctuated from 30.9 to 232 and no correlations were found between O:N ratios and SST or chlorophyll *a* standing stock in the water column [21]. We found that the O:N ratio of *E*. *pacifica* was 17.3–23.8 in March in the Yellow Sea, during a time of spawning activity[16], high grazing rate, and rich food supply. During September and December, the O:N ratio of *E*. *pacifica* was only 1.6–2.7 and 2.0–5.1 with low phytoplankton concentrations. The lower O:N ratio was a consequence of reduced respiration rates. According to the body carbon content of *E*. *pacifica*, more lipids were stored in March. Energy sources of *E*. *pacifica* in September and December are microzooplankton. The marked seasonal cycle in the O:N ratio appears to reflect the reproductive condition of *E*. *pacifica*, as no spawning occurred in August, September, October, or December and gravid females were first collected and spawned in March 2007 [16]. O:N ratios of *E*. *pacifica* in the Yellow Sea in March, September and December were negatively correlated with SSTs and no significant correlation was found between the O:N ratios and chlorophyll *a* concentrations. Seawater temperature is clearly the most important parameter influencing the metabolism of *E*. *pacifica*, which is obviously different to other sea areas, such as the Oyashio region.

### Status of *Euphausia pacifica* in the Yellow Sea bottom cold water

Iguchi and Ikeda showed that 11.4°C was the optimum temperature for *E*. *pacifica* growth in the Southern Japan Sea and it could not live in water over 20°C for more than 1 day [20]. High summer temperatures may suppress growth of *E*. *pacifica* changing the balance of parameters in metabolic responses [9]. During summer and autumn, surface seawater temperatures were > 20°C. *Euphausia pacifica* lived in the YSBCW and had lower grazing, metabolism, and reproduction rates. Egg production experiments were simultaneously conducted on the same cruises and have been reported in another paper [16]. In the egg production experiments, a total of 181 gravid females were incubated on board. Gravid *E*. *pacifica* females were first collected in March 2007, when 76.5% of the gravid females spawned and the average brood size was 71.33 eggs female^-1^. No gravid females were collected from August to December and no spawning occurred in August, September, or December. The spawning season for *E*. *pacifica* in the southern Yellow Sea apparently ends between late June and early August. The YSBCW and bottom layer of the thermocline could supply the necessary growth requirements for *E*. *pacifica*. Grazing behavior, energy consumption, body length and population structure suggest that the oversummering *E*. *pacifica* in the YSBCW were in diapause during summer and autumn.
